# Accurate, Fast and Cost-Effective Diagnostic Test for Monosomy 1p36 Using Real-Time Quantitative PCR

**DOI:** 10.1155/2014/836082

**Published:** 2014-04-15

**Authors:** Pricila da Silva Cunha, Heloisa B. Pena, Carla Sustek D'Angelo, Celia P. Koiffmann, Jill A. Rosenfeld, Lisa G. Shaffer, Martin Stofanko, Higgor Gonçalves-Dornelas, Sérgio Danilo Junho Pena

**Affiliations:** ^1^Departamento de Bioquímica e Imunologia, Instituto de Ciências Biológicas, Universidade Federal de Minas Gerais, Belo Horizonte 31270-901, MG, Brazil; ^2^GENE-Núcleo de Genética Médica, Avenida Afonso Pena 3111, 9th Floor, Belo Horizonte 30130-909, MG, Brazil; ^3^Departamento de Genética e Biologia Evolutiva, Instituto de Biociências, Universidade de São Paulo, São Paulo 05508-900, SP, Brazil; ^4^Signature Genomic Laboratories, PerkinElmer, Inc., Spokane, WA 99207, USA; ^5^Paw Print Genetics, Genetic Veterinary Sciences, Inc., Spokane, WA 99202, USA

## Abstract

Monosomy 1p36 is considered the most common subtelomeric deletion syndrome in humans and it accounts for 0.5–0.7% of all the cases of idiopathic intellectual disability. The molecular diagnosis is often made by microarray-based comparative genomic hybridization (aCGH), which has the drawback of being a high-cost technique. However, patients with classic monosomy 1p36 share some typical clinical characteristics that, together with its common prevalence, justify the development of a less expensive, targeted diagnostic method. In this study, we developed a simple, rapid, and inexpensive real-time quantitative PCR (qPCR) assay for targeted diagnosis of monosomy 1p36, easily accessible for low-budget laboratories in developing countries. For this, we have chosen two target genes which are deleted in the majority of patients with monosomy 1p36: *PRKCZ* and *SKI*. In total, 39 patients previously diagnosed with monosomy 1p36 by aCGH, fluorescent *in situ* hybridization (FISH), and/or multiplex ligation-dependent probe amplification (MLPA) all tested positive on our qPCR assay. By simultaneously using these two genes we have been able to detect 1p36 deletions with 100% sensitivity and 100% specificity. We conclude that qPCR of *PRKCZ* and *SKI* is a fast and accurate diagnostic test for monosomy 1p36, costing less than 10 US dollars in reagent costs.

## 1. Introduction

Monosomy 1p36 (OMIM #607872) is one of the most common chromosome abnormalities in humans, affecting approximately 1 in 5,000 live births. It is considered the most common subtelomeric deletion syndrome in humans, resulting from a heterozygous deletion of the most distal chromosomal band on the short arm of chromosome 1 [1–3]. Monosomy 1p36 is generally sporadic [2] and is believed to account for 0.5–0.7% of all cases of idiopathic intellectual disability. Several mechanisms may be involved in the generation and/or stabilization of the rearrangements in the 1p36 region, which may include terminal and interstitial deletions, derivative chromosomes, and more complex rearrangements, but the major mechanism stabilizing terminal deletions appears to be breakage-fusion-bridge (BFB) cycles [4]. There are no common breakpoints and the deletion sizes vary from <1 Mb to >10 Mb [2, 3, 5–8]. The majority of the patients with monosomy 1p36 have large terminal deletions [3], generally found within the first 4-5 Mb from the 1p telomere [2, 5, 7].

Patients with classic monosomy 1p36, who represent the majority of cases reported in the literature, have some typical clinical characteristics, with a phenotype that has been incorporated into standard malformation syndrome atlases [9]. It involves developmental delay and intellectual disability, both generally severe to profound, hypotonia and feeding problems in infancy and characteristic dysmorphic facial features which include: microcephaly, large late-closing anterior fontanelle, deep-set eyes, broad nasal bridge, straight eyebrows, and pointed chin [2, 9, 10]. Other features in some individuals include seizures, hearing loss, structural heart defects, cardiomyopathy, and behavior abnormalities. Some recent studies have reported the identification of a small proportion of patients with atypical “expanded” 1p36 phenotype, making the differential diagnosis difficult [11, 12]; however, according to Giannikou et al. [12], in these cases, the coexistence of additional “copy number variants” (CNVs) elsewhere in the genome may affect and explain partially or completely the variability of their clinical phenotype.

The laboratory validation of the clinical diagnosis is essential for establishing a medical prognosis and providing genetic counseling to the family. Since many 1p36 deletions are not able to be visualized by light microscopy, the molecular confirmation nowadays is most often achieved by microarray-based comparative genomic hybridization (aCGH). However, the high cost of this test limits its broad adoption, especially in developing countries [13].

Since a characteristic phenotype has been defined, monosomy 1p36 is amenable to targeted molecular diagnosis. For that, we felt that real-time quantitative PCR (qPCR) met the requirements of cost-effectiveness and easy execution [13–18]. Thus, we report the development of a sensitive, rapid, and affordable qPCR diagnostic method for monosomy 1p36, using two target genes which are deleted in the majority of patients with monosomy 1p36:* PRKCZ* and* SKI*. Using such double-pronged methodology we have been able to detect, with 100% sensitivity and 100% specificity, the 1p36 deletion in 39 patients who had been previously diagnosed by aCGH, fluorescent* in situ* hybridization (FISH), and/or multiplex ligation-dependent probe amplification (MLPA).

## 2. Material and Methods

### 2.1. Subjects

We studied 39 patients previously diagnosed with monosomy 1p36 (“Positive Group”; 11 males and 28 females). Twenty-eight patients (7 males and 21 females) had the diagnosis confirmed by aCGH at Signature Genomic Laboratories (Spokane, WA, USA). Some samples were tested by whole-genome, bacterial artificial chromosome-based microarray (SignatureChipWG, Signature Genomic Laboratories, Spokane, WA, USA) according to previously described methods [19], while others were analyzed by whole-genome, oligonucleotide-based microarrays custom-designed by Signature Genomics (SignatureChipOS, either version 1, a 105K-feature array manufactured by Agilent Technologies, Santa Clara, CA, USA, or version 2, a 135K-feature array manufactured by Roche NimbleGen, Madison, WI, USA) according to previously described methods [20, 21]. Ten patients (4 males and 6 females) were diagnosed using the techniques of FISH and/or MLPA, according to previously described methods [8], with addition of SALSA MLPA kit P070 Human Telomere-5 probemix (MRC-Holland, http://www.mlpa.com/). This step was carried out at Unidade de Aconselhamento Genético of the Centro de Estudos do Genoma Humano of Universidade de São Paulo (Instituto de Biociências, Departamento de Genética e Biologia Evolutiva, USP). One patient (male) was commercially diagnosed by aCGH at the Cytogenetics and Molecular Diagnostics Laboratory (University of Miami, Miami, FL, USA). As controls, we ran DNA from 50 normal patients (“Normal Controls”; 25 males and 25 females) from GENE-Núcleo de Genética Médica.

### 2.2. Ethics Statement

A written consent, approved by the Ethics Committee on Research on Humans of the Instituto de Biociências of Universidade de São Paulo (CEP-IB-USP), was obtained from the guardians of ten patients with monosomy 1p36 for using their genetic material. Two patients with monosomy 1p36 were enrolled in a research study on chromosome abnormalities approved by the Institutional Review Board Spokane. DNA samples from other participants with monosomy 1p36 were obtained from diagnostic procedures prior and anonymized prior to research use, without requiring specific ethics committee approval. This study was conducted in accordance with the principles of the Declaration of Helsinki.

### 2.3. Real-Time Quantitative PCR (qPCR)

The qPCR assays for monosomy 1p36 were performed with two target genes:* PRKCZ* (protein kinase C, zeta) and* SKI* (v-ski avian sarcoma viral oncogene homolog). The primers used for amplification of both markers were designed using the Primer3 program, version 0.4.0 (http://frodo.wi.mit.edu/). The primer sequences were aligned against the entire human genome using the UCSC program (http://www.genome.ucsc.edu/). This step was performed to ensure that the primers amplified only the genomic region of interest and also to guarantee that the forward and reverse primers were free of single nucleotide polymorphisms (SNPs). The* HMBS* marker (hydroxymethylbilane synthase) was chosen as reference gene, and the primer sequences used for its amplification were derived from Saugier-Veber et al. [22], without the addition of the universal extension cited by the authors. The primer sequences and the sizes of the amplicons are shown in Table 1.

Each qPCR reaction contained 5 *μ*L of SYBR Green PCR Master Mix 2X (Applied Biosystems, Foster City, CA, USA); 10 ng of genomic DNA; forward and reverse primers at optimized concentrations; and sterile water up to a final volume of 10 *μ*L. The reaction profile was an initial step of 50°C for 2 min and a step of denaturation at 95°C for 10 min, followed by 50 cycles of denaturation at 95°C for 15 sec and combined annealing and extension at 60°C for 60 sec. All the samples were subjected to gradual denaturation to determine the melting curve after 50 amplification cycles. A “no template control” (NTC) was made in all the qPCR reactions for each pair of primers containing all the reagents except DNA. The qPCR reactions were performed using Rotor-Gene Q (Qiagen Inc, Valencia, CA, USA) equipment, and the data were processed by the associated Rotor-Gene Q Series Software, version 1.7 (build 94) (Qiagen Inc, Valencia, CA, USA).

The optimum concentration of each primer was determined by an initial test called concentration test of primers. The concentrations tested were 0.1, 0.2, 0.3, 0.4, and 0.6 *μ*M. From this test, the concentrations of each forward (F) and reverse (R) primer to be used in qPCR reactions were standardized in 0.1 *μ*M (F)/0.1 *μ*M (R) for* HMBS *and* SKI* and 0.2 *μ*M (F)/0.2 *μ*M (R) for* PRKCZ*.

Standard curves for all the primer sets were generated with series of log dilution of genomic DNA: 20, 10, 5, and 2.5 nM for* HMBS *and 10^1^, 10^0^, 10^−1^, and 10^−2 ^nM for* PRKCZ *and* SKI*. Each dilution was tested in triplicate. Slopes derived from standard curves were used to calculate the efficiency of the qPCR reaction for each marker and also to normalize qPCR data. Reaction specificity was confirmed with melting curves analysis and polyacrylamide gel electrophoresis experiments.

A negative control sample was used in all the qPCR runs. This negative control corresponds to the DNA of an individual who is not affected by monosomy 1p36. The presence of the negative control in each qPCR run is fundamental for the final calculation of copy number change of each marker.

### 2.4. Analysis of the qPCR Data

The equations used for normalization of the qPCR data and for the calculation of the allelic copy number of each marker were derived from studies of Weksberg et al. [13] and Hughes et al. [17]. The D'Agostino-Pearson test for normal distribution of the values of fold copy number change (ΔKC_t_) and ROC curve (receiver operating characteristic curve) analyzes were made using MedCalc software, version 12.2.1 (MedCalc Software, Mariakerke, Belgium). The measures used to assess the accuracy of qPCR in discriminating between the group of normal controls and the positive group were sensitivity, specificity, and area under the ROC curve (AUC). For each marker a dot plot was also obtained using MedCalc software.

## 3. Results

**Figure 1:**
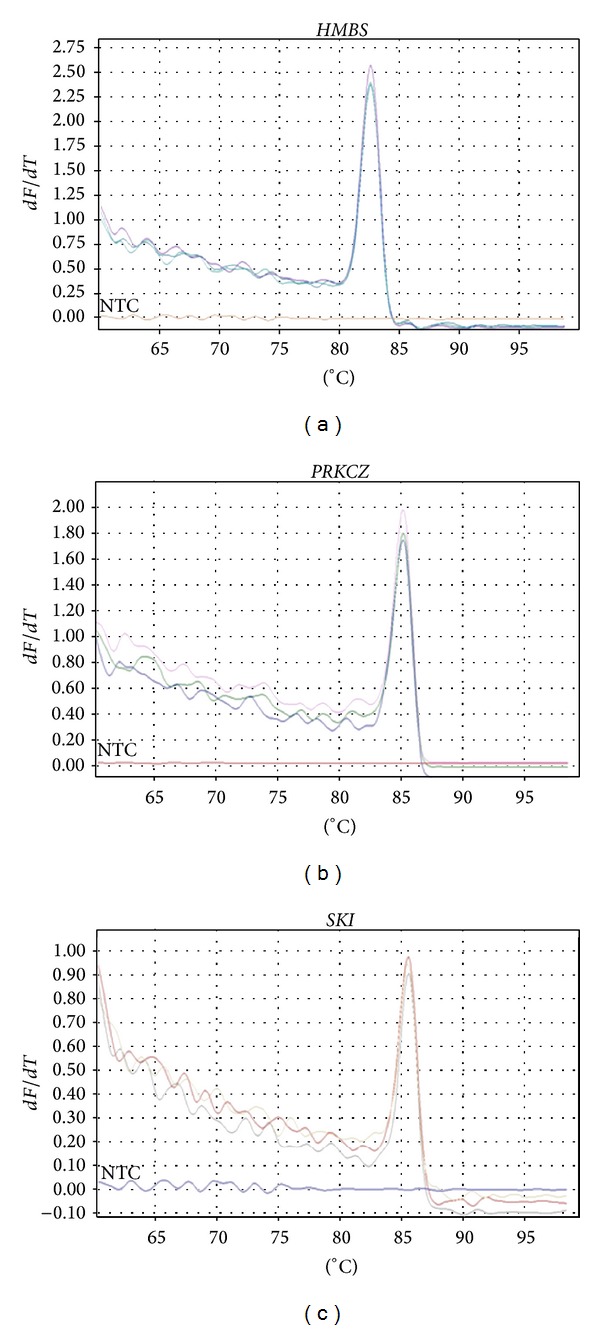
Melting curve for the markers: (a)* HMBS*, (b)* PRKCZ,* and (c)* SKI*. All the markers showed the presence of a single dissociation peak and absence of primer dimers. The melting temperature of each amplicon was:* HMBS*: *T*_*m*_ = 82.6°C;* PRKCZ*: *T*_*m*_ = 85.2°C;* SKI*: *T*_*m*_ = 85.6°C. NTC: No template control. These results were obtained using the Rotor-Gene Q Series Software, version 1.7 (build 94) (Qiagen Inc., Valencia, CA, USA).

Polyacrylamide gel electrophoresis experiments (data not shown) and melting curve analyzes confirmed the specificity of the qPCR assays for amplification of the reference gene (*HMBS*) and of the two target genes (*PRKCZ *and* SKI*) (see Figures 1(a), 1(b), and 1(c) for melting curve of, resp.,* HMBS*,* PRKCZ,* and* SKI*).

The qPCR reactions occurred with high efficiency (greater than 94%), with R^2^ values of the standard curve greater than 0.99 (Table 2).

The calculation of ΔKC_t_ for* PRKCZ* and* SKI *was performed considering the groups “Normal Controls” (Supplementary Table 1, available online at http://dx.doi.org/10.1155/2014/836082) and “Positive Group” (Supplementary Table 2).  Table 3 shows an overview of the ΔKC_t_ results for the two above groups. The value of the mean ΔKC_t_ ± SD for the normal controls was near zero for both markers:* PRKCZ* (−0.0612 ± 0.1554) and* SKI *(−0.1264 ± 0.1401), indicating the presence of two allelic copies of each marker (Table 3).

The value of the mean ΔKC_t_  ± SD for the positive group was near −1.000 for the two markers:* PRKCZ *(−0.9696 ± 0.1649) and* SKI* (−1.0287 ± 0.2880) (Table 3). The maximum value of ΔKC_t_ for* PRKCZ* in the positive group (−0.6676) was lower than the minimum value of ΔKC_t_ obtained in the group of normal controls (−0.3661), showing a complete separation of* PRKCZ* results between the two groups (Table 3). These results show that the 39 patients have hemizygous microdeletion of* PRKCZ*, which in turn is sufficient to identify them as having monosomy 1p36.

In the analysis of the* SKI* marker, one of the patients (subject 11 of Supplementary Table 2) showed a ΔKC_t_ value near zero (−0.2876), corresponding to the maximum value of ΔKC_t_ obtained for this marker in the positive group (Table 3). This result of ΔKC_t_ was located in the normal range for the* SKI* marker (Table 3). However, this patient presented clear hemizygosity of* PRKCZ* (ΔKC_t_ = −1.2278), confirming the presence of monosomy 1p36 (Supplementary Table 2). Equal results had been previously obtained by MLPA (data not shown). ΔKC_t_ results of the* SKI* marker for the remaining 38 patients (−0.5479 to −1.5803) were lower than the minimum value of ΔKC_t_ obtained in the group of normal controls (−0.3794), showing a complete separation of* SKI* results between the 38 patients and the 50 normal controls (Table 3).

The results described above were corroborated by statistical analyzes made using the MedCalc software, version 12.2.1 (MedCalc Software, Mariakerke, Belgium). The ΔKC_t_ values for both markers in the group of normal controls presented normal distribution according to D'Agostino-Pearson test (data not shown). The detection performance of the primers was evaluated using ROC curve analyzes.

**Figure 2:**
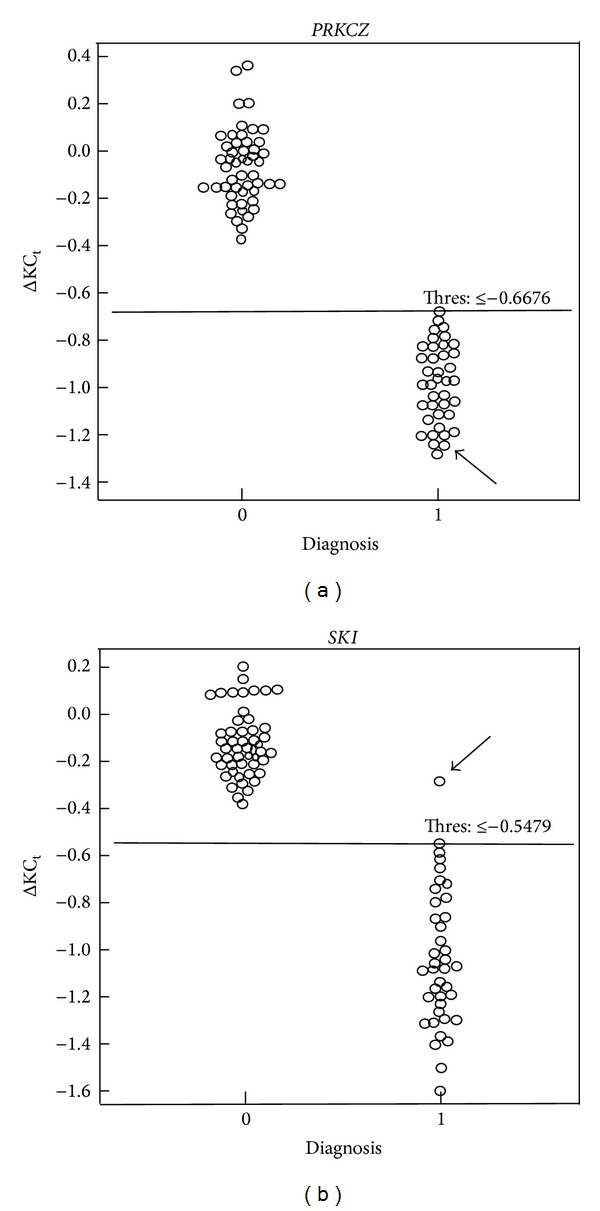
Dot plot for the markers which were analyzed in the study of monosomy 1p36: (a)* PRKCZ* and (b)* SKI*. ΔKC_t_ (fold copy number change) values of the 50 normal controls and of the 39 patients with monosomy 1p36 were considered for construction of the dot plot. The threshold value (Thres) is shown inside of each graph. On the horizontal axis (Diagnosis), the number 0 represents the negative group (normal controls) and the number 1 represents the positive group. Each individual is represented by a circle within the graph. The horizontal line within the graph indicates the threshold value, which corresponds to the ΔKC_t_ value chosen as limit of separation between the two groups. ΔKC_t_ result obtained for patient 11 is indicated by a black arrow in both graphs. This patient does not have deletion of* SKI* (ΔKC_t_ = −0.2876), but he presents hemizygous deletion of* PRKCZ* (ΔKC_t_ = −1.2278), confirming that he has monosomy 1p36. Equal results were previously obtained by MLPA. The simultaneous use of these two genes in the qPCR assays resulted in a test with 100% sensitivity and 100% specificity. The graphs were obtained using MedCalc software, version 12.2.1 (MedCalc Software, Mariakerke, Belgium).

Using the ΔKC_t_ values of the negative (normal controls) and positive groups (Supplementary Tables 1 and 2) and considering that the qPCR approach of this study is a double-pronged methodology, the simultaneous use of* PRKCZ* and* SKI* markers in qPCR assays produced a ROC curve with AUC equal to 1.000 (95% CI, 0.959–1.000) and corresponding *P* value equal to zero. This confirms that the qPCR technique was capable to differentiate between an individual who has a hemizygous microdeletion in 1p36 region and a normal individual. And by using these two genes we achieved a test with 100% sensitivity (95% CI, 91.0–100.0) and 100% specificity (95% CI, 92.9–100.0) (Figure 2). The threshold value for each marker, which was chosen as the limit of separation between the positive and negative groups, is shown in Figure 2.

All results obtained for monosomy 1p36 using the qPCR technique were also previously observed using other methods of analyzes (aCGH, FISH, and MLPA). For the majority of the patients it was possible to determine that both* PRKCZ* and* SKI* genes were deleted using the techniques described above. In the specific case of patient 10 there was a difference in the results obtained for the* SKI* gene specifically. According to previous MLPA data, patient 10 had an unbalanced translocation between subtelomeric regions of 1p and 7q, presenting a 1p36 deletion of ~1.9–2.2 Mb and a 7q duplication, the size of which has not been determined (data not shown). Moreover, the MLPA analysis showed that the deletion breakpoint in this patient occurred somewhere within the 281 kb interval, between the genomic coordinates chr1:1,956,418-2,237,544, according to UCSC Genome Browser, Human February 2009 Assembly (GRCh37/hg19; http://www.genome.ucsc.edu/). Of note, the MLPA probe specific for the* SKI *gene remained intact in this patient, while the qPCR results showed deletion of both* PRKCZ* (ΔKC_t_ = −1.1885) and* SKI* (ΔKC_t_ = −1.0710) genes (Supplementary Table 2). The probable explanation for this divergence is that the MLPA probe and the qPCR primers map to different regions within the* SKI *gene. The* SKI* gene is mapped between genomic coordinates chr1:2,160,134-2,241,652 (UCSC Genome Browser, Human February 2009 Assembly); the MLPA probe is mapped between coordinates chr1:2,237,544-2,237,607 (UCSC Genome Browser, Human February 2009 Assembly) and the qPCR primers are mapped between coordinates chr1:2,221,777-2,221,861 (UCSC Genome Browser, Human February 2009 Assembly). Thus, knowing that this patient presented deletion of ten 1p36 probes distal to the* SKI* probe (data not shown) and that the qPCR primers are also located distal to this probe, it is likely that patient 10 has part of the* SKI* gene deleted (region of location of the qPCR primers) and another part intact (region of location of the MLPA probe).

From our data it is clear that the qPCR technique using the two genes,* PRKCZ* and* SKI*, was efficient and accurate for detection of microdeletions associated with monosomy 1p36.

## 4. Discussion

About 3% of the world population has intellectual disability, 20% to 50% of which is caused by chromosome abnormalities [23]. One of the most common chromosome abnormalities in humans is monosomy 1p36 (OMIM #607872), and it accounts for 0.5–0.7% of all cases of idiopathic intellectual disability [24]. The confirmation of clinical suspicion is essential for clinical monitoring of the patient and genetic counseling of the family.

Medical genetics laboratories generally use aCGH for the diagnosis of monosomy 1p36. However, the high cost of this test limits its broad adoption, especially in developing countries [13]. In face of this reality, we found in qPCR technique the requirements of cost-effectiveness and easy execution for targeted diagnosis of monosomy 1p36, easily accessible for low-budget laboratories in developing countries [13–18]. Thus, in this study, we report the development of a qPCR assay for the detection of copy number changes in the 1p36 region using two target genes,* PRKCZ* and* SKI*, which are deleted in the majority of patients with monosomy 1p36.

The haploinsufficiency of* PRKCZ* and* SKI* genes has been proposed to be related to the neurologic phenotype seen in patients with monosomy 1p36, thus contributing to the neurodevelopmental delay [3, 8, 25, 26]. As this feature is observed in all the patients with monosomy 1p36 [5, 10, 27, 28], we can suggest that these two genes will very likely be deleted in a majority of patients.

*PRKCZ* and* SKI* are located ~2.0 Mb (chr1:1,981,909-2,116,834) and ~2.2 Mb (chr1:2,160,134-2,241,652), respectively, from the 1p telomere (UCSC Genome Browser, Human February 2009 Assembly), and previous studies have shown that both genes are within the region where the majority of 1p36 deletions occur.

In one of the first large studies related to monosomy 1p36, Heilstedt et al. [5] evaluated the deletion sizes in 61 affected subjects from 60 families and noticed that, although the deletion sizes ranged widely, 12.5% of breakpoints clustered 4.0–4.5 Mb from the telomere, and 40% of all the breakpoints occurred 3.0–5.0 Mb from the telomere. In a study involving subtelomeric abnormalities, Ballif et al. [7] analyzed 32 positive individuals for monosomy 1p36 and found that 54% of the breakpoints were located within the first 5.0 Mb from the 1p telomere and ~90% were located within the first 10 Mb. D'Angelo et al. [8] detected a large variability in the sizes of deletions (~2.0–10 Mb) when they analyzed nine patients; however, all the patients showed deletion of* PRKCZ* and* SKI*.

In other studies, performed with a smaller number of patients, there was also a change in the number of copies of* PRKCZ* and* SKI *in most of affected individuals, which support our results. Gajecka et al. [29] characterized complex rearrangements in four individuals with deletions, duplications, and/or triplications of 1p36 and they compared the regions of imbalance to two cases published. Results of aCGH and FISH revealed an overlapping region of 1.1 Mb containing* PRKCZ* and* SKI*, which were deleted in four individuals and duplicated/triplicated in two individuals. Vieira et al. [30] used aCGH to evaluate one patient that, despite showing a Smith-Magenis-like phenotype, lacked the 17p11.2 deletion or a mutation in* RAI1*. They detected a deletion of approximately 2.15 Mb in 1p36.32-1p36.33 region containing* PRKCZ* and* SKI*, which resulted in the final diagnosis of monosomy 1p36.

Rosenfeld et al. [3] characterized small interstitial deletions, and they reported five patients with 199–823 kb overlapping deletions of proximal 1p36.33, three of which included* PRKCZ* and* SKI*. A small interstitial deletion (1.52 Mb) including* PRKCZ* and* SKI* was found by Gajecka et al. [31] during evaluation of two siblings with mild phenotypic features of monosomy 1p36. It is important to emphasize that most of the genes which were contained in the smallest region of deletion (174 kb) characterized by Rosenfeld et al. [3] were not deleted in the patients analyzed by Gajecka et al. [31], with only one of a total of five genes showing partial deletion and the others remaining intact. Indeed the specific critical region for monosomy 1p36 has not been determined yet, greatly making its study difficult. Really, there does not seem to be a single critical region for this syndrome.

The qPCR results showed that both markers were efficient in the identification of patients with 1p36 microdeletions, resulting in a test with 100% sensitivity and 100% specificity. With the exception of the result of patient 10 for* SKI* marker (subject 10 of Supplementary Table 2), all the qPCR results were 100% concordant with results previously determined by aCGH, FISH, and/or MLPA. It is important highlight that rare patients, who have mainly small and atypical interstitial microdeletions, might, occasionally, not be identified by this qPCR assay. However, the qPCR assay presented here enables the identification of the majority of patients with 1p36 monosomy.

We have calculated that detecting a patient suspected of having monosomy 1p36 by qPCR would have a final cost of US$ 8.13. This calculation was done considering only the necessary reagents for the qPCR reaction. The costs involving the reagents of other steps, such as DNA extraction, and the costs of laboratory materials, equipment, and workforce were not included in this calculation. Moreover, the whole screening process from the genomic DNA extraction to the analysis of the qPCR data could be performed in a short time (about 8 hours).

In summary, the results presented here have proven that qPCR of* PRKCZ* and* SKI* can be a fast, accurate, and cost-effective diagnostic test for monosomy 1p36.

## 5. Conclusions

Here we report the development of a simple, rapid, and inexpensive real-time quantitative PCR (qPCR) assay for targeted diagnosis of monosomy 1p36. We showed that qPCR of* PRKCZ* and* SKI* can be considered an accurate diagnostic test for monosomy 1p36, easily accessible for low-budget laboratories in developing countries.

## Supplementary Material

Supplementary Table 1: includes the *δ*KCt results of PRKCZ and SKI markers for 50 normal controls, Supplementary Table 2: includes the *δ*KCt results of PRKCZ and SKI markers for 39 patients with monosomy 1p36.

## Figures and Tables

**Table 1:** qPCR primer sequences, genomic location, and size of each amplicon.

Marker	Chr	Primer name	Sequence (5′ → 3′)	Amplicon size	Genomic location of amplicon (hg19)
*PRKCZ* ^ a^	1	PRKCZ-F	ACGGTGTGAGCATGAGGATAC^c^	125	2,020,732-2,020,856
		PRKCZ-R	CAGAGGCTGAAGCAAATGAAC^c^		

*SKI* ^ a^	1	SKI-F	AGCTGATTGGGGGTAGGC^c^	85	2,221,777-2,221,861
		SKI-R	TCAGGCTGAGCAGTGCAG^c^		

*HMBS* ^ b^	11	HMBS-F	ACGGCTCAGATAGCATACAAG^d^	185	118,963,676-118,963,860
		HMBS-R	ATGCCTACCAACTGTGGGTCA^d^		

Chr: chromosome; ^a^target gene; ^b^reference gene; ^c^primer sequences designed using Primer3 software, version 0.4.0 (http://frodo.wi.mit.edu/); ^d^primer sequences derived from Saugier-Veber et al. [22].

**Table 2:** Slope, amplification efficiency, and R^2^ values for all the markers.

Marker	Slope	Amplification efficiency	R^2^
*PRKCZ* ^ a^	−3.1950	94.42%	0.9981
*SKI* ^ a^	−3.1910	94.23%	0.9975
*HMBS* ^ b^	−3.4350	95.49%	0.9964

R^2^: determination coefficient; ^a^target gene; ^b^reference gene.

**Table 3:** Overview of the qPCR markers results.

Sample types	Marker	*n*	ΔKC_t_				
			Mean	SD	Median	Maximum	Minimum
Normal controls	*PRKCZ *	50	−0.0612	0.1554	−0.0533	0.3551	−0.3661
	*SKI *	50	−0.1264	0.1401	−0.1514	0.1956	−0.3794

Positive group	*PRKCZ *	39	**−0.9696**	**0.1649**	−0.9634	−0.6676	−1.2644
	*SKI *	39	**−1.0287**	**0.2880**	−1.0710	−0.2876^a^	−1.5803

ΔKC_t_: fold copy number change; *n*: sample size; SD: standard deviation.

ΔKC_t_ results (expressed as mean ΔKC_t_  ± SD) consistent with the loss of one allelic copy are indicated in bold.

^a^ΔKC_t_ result obtained for patient 11 (subject 11 of Supplementary Table  2), indicative of nondeletion of *SKI* gene. This patient presented hemizygosity of *PRKCZ* (ΔKC_t_ = − 1.2278), confirming that he has monosomy 1p36. Equal results were obtained by MLPA (data not shown). The remaining 38 patients had ΔKC_t_ results corresponding to hemizygous microdeletion of *SKI* (−0.5479 to −1.5803) and *PRKCZ* (−0.6676 to −1.2644).
