# Supplementary material for: Accurate, Fast and Cost-Effective Diagnostic Test for Monosomy 1p36 Using Real-Time Quantitative PCR
Source: Dis Markers. 2014 Apr 15;2014:836082. doi: 10.1155/2014/836082 (PMC4009252; doi:10.1155/2014/836082)
Supplement: Supplementary file 1 — Supplementary Table 1: includes the δKCt results of PRKCZ and SKI markers for 50 normal controls, Supplementary Table 2: includes the δKCt results of PRKCZ and SKI markers for 39 patients with monosomy 1p36. [file 836082.f1.pdf]

Supplementary TABLE 1 ΔKC_t results for 50 normal controls

		ΔKC_t	
**Sample type**	**Subjects**	***PRKCZ***	***SKI***
Normal Controls	1	-0.0074	-0.2087
	2	-0.0467	-0.1680
	3	0.0793	0.0978
	4	0.0923	-0.1688
	5	-0.1521	-0.0831
	6	-0.0198	0.0886
	7	0.0035	0.0921
	8	-0.1814	-0.0329
	9	-0.2216	-0.1132
	10	0.0379	-0.2697
	11	-0.2022	-0.1232
	12	0.3551	-0.2088
	13	-0.0598	-0.0730
	14	-0.1319	-0.1181
	15	-0.1492	-0.2148
	16	-0.2138	-0.0623
	17	0.2010	-0.2487
	18	0.3441	0.1956
	19	-0.0950	-0.3521
	20	-0.1465	-0.2946
	21	-0.1079	-0.2855
	22	-0.0241	-0.2563
	23	-0.0348	-0.2170
	24	-0.2366	-0.1446
	25	0.0103	-0.1026
	26	-0.2395	-0.0786
	27	-0.3661	-0.2200
	28	-0.2513	-0.0660
	29	-0.3256	-0.3794
	30	0.0228	-0.3301
	31	-0.2923	-0.2553
	32	-0.1458	-0.3089
	33	-0.0347	-0.1881
	34	-0.1632	-0.1509
	35	-0.0198	0.0069
	36	-0.2690	-0.1764
	37	0.2061	-0.1931
	38	-0.0330	-0.1283
	39	-0.1300	-0.1682
	40	0.0382	0.0818
	41	0.0733	-0.2126
	42	-0.0118	0.1018
	43	0.1093	0.0932
	44	-0.0951	-0.2411
	45	-0.1358	-0.1519
	46	-0.1325	-0.1884
	47	0.0384	-0.0276
	48	0.0707	0.1046
	49	0.0891	0.1431
	50	-0.1565	-0.1126

ΔKC_t: fold copy number change.

Supplementary TABLE 2. ΔKC_t results for 39 patients with monosomy 1p36

		ΔKC_t	
**Sample type**	**Subjects**	***PRKCZ***	***SKI***
	1	-1.0444	-1.5803
	2	-0.9740	-1.0794
	3	-0.9203	-1.1887
	4	-1.2644	-1.2929
	5	-1.1825	-1.4869
	6	-1.1014	-1.1596
	7	-1.2279	-1.3524
	8	-1.1512	-1.3902
	9	-1.0553	-1.2166
	10	-1.1885	-1.0710
	11	-1.2278	**-0.2876**
	12	-0.9493	-1.3007
	13	-0.7394	-1.0284
	14	-0.8178	-0.8545
	15	-0.9758	-1.3748
	16	-1.1804	-0.8959
	17	-0.7972	-0.8640
	18	-0.8646	-1.2867
	19	-0.7494	-1.1506
**Positive Group**	20	-0.8139	-0.7105
	21	-0.8971	-0.5823
	22	-0.7758	-0.7017
	23	-0.7976	-0.7339
	24	-0.7712	-0.9568
	25	-0.6676	-0.7665
	26	-0.8649	-1.0072
	27	-1.0624	-1.0569
	28	-0.8522	-1.0492
	29	-0.9593	-1.0733
	30	-0.9222	-1.1846
	31	-1.0196	-1.2841
	32	-1.0632	-1.1287
	33	-1.1011	-0.9986
	34	-0.9634	-1.1797
	35	-0.7056	-0.6125
	36	-0.8442	-0.5479
	37	-1.1256	-0.6454
	38	-1.1701	-0.7919
	39	-1.0243	-1.2477

ΔKC_t: fold copy number change. ΔKC_t result of the *SKI* marker for patient 11 is in bold and it indicates the presence of two allelic copies of *SKI* in this patient. This patient had hemizygous microdeletion of *PRKCZ* (ΔKC_t = -1.2278), confirming that he has monosomy 1p36. Equal results were obtained by MLPA (data not shown).
